# Thicknesses of Macular Inner Retinal Layers in Children with Anisometropic Amblyopia

**DOI:** 10.1155/2020/6853258

**Published:** 2020-10-19

**Authors:** Zheren Xia, Hao Chen, Suilian Zheng

**Affiliations:** Department of Ophthalmology, The Second Affiliated Hospital and Yuying Children's Hospital of Wenzhou Medical University, Wenzhou, China

## Abstract

**Objective:**

To investigate the thicknesses of macular inner retinal layers in children with anisometropic amblyopia using spectral domain optical coherence tomography (SD-OCT).

**Methods:**

Thirty-seven children with anisometropic amblyopia and fifty-seven children with normal vision were recruited in the study. Both eyes of children with anisometropic amblyopia and the right eyes of normal controls underwent scanning with the Spectralis OCT. The segmentation of retinal layers was performed automatically to measure individual inner retinal layers in the five sectors of the macular. An independent sample *t*-test was applied to compare the mean layer thicknesses of anisometropic eyes and fellow eyes with those of control eyes.

**Results:**

There was no significant difference in the total macular thickness between amblyopic and control eyes. However, in the peripheral macular area, three of the four quadrants of both the ganglion cell layer (GCL) and the inner plexiform layer (IPL) thicknesses were significantly reduced in amblyopic eyes compared to control eyes. Moreover, two of the four quadrants of the GCL thickness and three of the four quadrants of the IPL thickness in the peripheral macular area were significantly reduced in fellow eyes than in control eyes.

**Conclusion:**

The SD-OCT data revealed differences in the thicknesses of some macular inner retinal layers in both eyes of children with anisometropic amblyopia compared with those with emmetropia, indicating that structural changes might exist in the retina of children with amblyopia.

## 1. Introduction

Amblyopia is one of the most common visual disorders in children [1]. The main causes include strabismus, anisometropia, or an obstruction along the visual axis. In the study of the pathogenesis of amblyopia, it was concluded that amblyopia is a developmental disorder which includes a pathophysiological change from retinal ganglion cells to the visual cortex. There are two central [2, 3] and peripheral [4, 5] theories in this regard. Animal experiments and functional magnetic resonance imaging have confirmed the presence of histological changes in the hypothalamus in patients with amblyopia [2, 6]. However, because of technological limitations of assessment, the change of retinal structure is still controversial.

The Spectralis OCT (Heidelberg, Germany), based on spectral domain optical coherence tomography (SD-OCT) technology, has improved resolution that is capable of performing more precise measurements of retinal layers. Furthermore, it can provide automated segmentation and quantification of each retinal layer with a built-in software. Thus, investigators began to explore the changes in OCT images to verify the presence of retinal dysfunction. The changes of the inner retina, such as the ganglion cell layer (GCL) and retinal nerve fiber layer (RNFL), are applied to diagnose glaucoma, multiple sclerosis, anterior ischemic optic neuropathy, and other diseases [7–9].

This study is aimed at assessing alterations in the thicknesses of macular inner retinal layers in children with anisometropic amblyopia using the Spectralis OCT.

## 2. Material and Methods

Thirty-seven children with anisometropic amblyopia were recruited from July 2014 to February 2017 in the Ophthalmology Department, the Second Affiliated Hospital of Wenzhou Medical University, Wenzhou, China. Both eyes of each child were included in the study, which were served as the amblyopia eye group and the fellow eye group. The right eyes of 57 children with emmetropia were enrolled as control subjects.

All subjects were aged from 4 to 14 years. The amblyopic subjects were enrolled in this study with the best-corrected visual acuity (BCVA) between 20/32 and 20/400 in the amblyopic eye and 20/40 or better in the normal eye (age 4 to ≤5 years: visual acuity ≥ 20/40; age > 5: visual acuity ≥ 20/30), respectively. The intereye BCVA difference is ≥2 logarithm of the minimum angle of resolution lines (LogMAR). Anisometropia was defined as the difference of interocular spherical equivalent or astigmatism equal to or more than 1.0 diopter (*D*). The control subjects should have visual acuity equal to or greater than 20/20 in each eye and the refractive error ranging from −0.50 to +0.50 DS. Patients were excluded if they were not cooperative enough for OCT examination or combined with several conditions as follows: organic eye diseases such as cataract, glaucoma, retinal diseases, and strabismus.

All subjects underwent a thorough ophthalmic examination, including extraocular movements, visual acuity (Snellen E chart), slit-lamp biomicroscopy, intraocular pressure, fundus examination, and axial length (using Carl Zeiss IOL Master; Carl Zeiss AG, Oberkochen, Germany). Then, after 30 minutes of use of three drops of 1% cyclopentolate (Cyclogyl; Alcon Couvreur, Purrs, Belgium), cycloplegic refraction was performed with an autorefractometer.

All subjects gave their informed consent according to the Declaration of Helsinki (1964), and the study was approved by the Research Ethics Committee of the Second Hospital of Wenzhou Medical University. Informed consent was obtained from all individual participants (or legal parent or guardian for children) included in the study.

**Figure 1:**
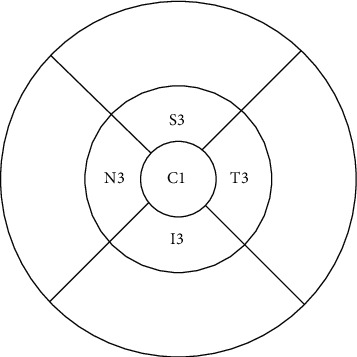
ETDRS grid showing C1, T3, I3, N3, and S3.

The thickness of each layer was identified and measured automatically by Spectralis OCT (Heidelberg Engineering, Heidelberg, Germany). The TruTrack active eye-tracking system was applied to increase scan quality. Volume scan mode was performed to obtain a 20° × 20° macular cube scan image (49 B-scan sections, 120 *μ*m spacing, and 512 A scans/B scans). Macular thickness was measured with the Early Treatment Diabetic Retinopathy Study (ETDRS) grid, which comprises three concentric circles with diameters of 1, 3, and 6 mm. For the reason of children's cooperation, we took the area only within the inner ring for analysis in order to ensure the reliability of the data. According to the manufacturer guidelines, only images that had higher than 25 dB (ranges from 0 to 40) of quality score were included. We also check the segmentation; the scan was excluded if errors were present (such as the lines not corresponding to the proper retinal layers). The parameters in the following five sectors were recorded in this study: C1 (the average thickness in the central 1 mm diameter) and N3/I3/T3/S3 (the average thickness in the nasal/inferior/temporal/superior quadrant of a concentric ring, with an inner diameter of 1 mm and outer diameter of 3 mm of the ETDRS grid) (Figure 1).

**Figure 2:**
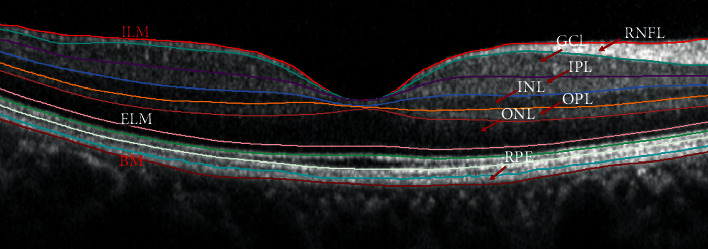
Retinal layer analysis performed by the segmentation software of the Spectralis.

The retina was segmented automatically into seven layers with a built-in software for the Spectralis OCT: (1) retinal never fiber layer (RNFL), (2) ganglion cell layer (GCL), (3) inner plexiform layer (IPL), (4) inner nuclear layer (INL), (5) outer plexiform layer (OPL), (6) outer nuclear layer (ONL), and (7) retinal pigment epithelium (RPE). From the map of retinal layer thickness, data were divided into five macular sectors. Each individual layer thicknesses of these five sectors of were registered. We chose the four inner retinal layers (RNFL, GCL, IPL, and INL) for analysis (Figure 2).

SPSS software version 22.0 (International Business Machines Corp., NY, USA) was applied for the statistical analysis of our data. Continuous data has been presented as the means ± standard deviations (SD). The distributions of age, VA of the amblyopic eye, VA of the fellow eye, spherical equivalent, axial length, and retinal thickness (total or each layer) were confirmed as normally distributed by the Kolmogorov–Smirnov test. Snellen visual acuities were transformed to LogMAR visual acuities for the statistical analysis. An independent sample *t*-test was used to compare refractive error, axial length, and mean layer thicknesses of anisometropic eyes and fellow eyes with control eyes, respectively. Meanwhile, the same statistical method was used to compare the ages between the anisometropic subjects and the control subjects. *P* values < 0.05 were considered statistically significant.

## 3. Results

The study included 37 children with anisometropic amblyopia (21 females, 16 males), with a mean age of 7.43 ± 2.62 (range: 4–14) years. Of these children, 16 had amblyopia in their right eye and 21 had it in their left eye. No significant difference was found in age between the amblyopic subjects and controls (*p* = 0.19). The mean spherical equivalent refractive error was +1.52 ± 3.42 (range: −4.38 to +7.50) *D* in amblyopic eyes and +0.21 ± 1.50 (range: −3.63 to +3.50) *D* in fellow eyes. There was a significant difference in the refractive error between amblyopic and control eyes (*p* < 0.01), as well as the difference between amblyopic and fellow eyes (*p* < 0.01). Both amblyopic and fellow eyes had no significant difference in the axial length of control eyes (*p* = 0.08 and 0.12). The mean BCVA in LogMAR was 0.40 ± 0.22 in amblyopic eyes, which was significantly different in fellow eyes (*p* < 0.01) and in control eyes (*p* < 0.01) (Table 1).

The total macular thickness (TMT) in C1 was 254.83 ± 23.73 *μ*m in amblyopic eyes, 253.48 ± 21.51 *μ*m in fellow eyes, and 255.91 ± 18.87 *μ*m in control eyes. There was no significant difference in TMT in the five sectors between amblyopic and control eyes, and only in T3, there was a significant difference between fellow and control eyes (*p* = 0.04) (Table 2).

The macular RNFL thickness did not differ significantly in the five sectors between amblyopic and control eyes. The GCL thickness measurements in S3, T3, and N3 were significantly reduced between amblyopic and fellow eyes. All measurements, except C1 and T3 of the IPL thickness, were significantly reduced in amblyopic eyes compared with control eyes. There was a significant difference only in C1 in the INL thickness measurements between amblyopic and control eyes (Table 3).

No significant differences were noted between fellow and control eyes in the RNFL and INL thicknesses. The GCL thickness measurements in S3 and T3 were significantly reduced in fellow eyes. The IPL thickness measurements in S3, T3, and N3 were significantly decreased in fellow eyes as compared to control eyes (Table 3).

All the findings and *P* values are summarized in Tables 2 and 3.

## 4. Discussion

Bilateral alterations of inner retinal thickness in children with anisometropic amblyopia were found according to our study. The GCL and IPL thicknesses of amblyopic eyes in the three areas of peripheral macular were smaller than those of control eyes, indicating that structural differences might exist in amblyopic eyes. Similar to our finding, Kyung-Ah et al. [10] evaluated that the thickness of the peripheral macular areas of eyes with unilateral amblyopia, significant thinning of GCL + IPL in amblyopic eyes was found. They believe that it may be related to the changes in the retinal microstructure such as degeneration of retinal ganglion cells, a reduction in the number of bipolar synapses in the IPL, and a decrease in the density of Müller fibers, which were demonstrated by some animal studies. However, in the central area, the thickness of both two layers in amblyopic eyes had no difference with that in control eyes. The results were consistent with the study of Chen et al. [11], which analyzed the thickness of each retinal layer at the foveal center and 0.5 mm from the foveal center in four directions. It is possible that different macular locations undergo different structural changes. Further research with a larger sample size is necessary to clarify this.

IPL is the second synaptic layer of the retina. The synapses in bipolar, amacrine, and ganglion cells in IPL are involved in the construction of a complex visual signal processing network. Ji et al. [12] found that the P1 wave amplitude density of multifocal electroretinography (mfERG) first-order kernel in amblyopic eyes was significantly attenuated compared with that in control eyes, which may reflect the abnormality of the retinal nerve in bipolar cell function and visual information transmission. While the relationship between the retinal structure and function of amblyopia remains a question, Betul et al. [13] reported a significant reduction of pattern electroretinogram (PERG) amplitude in amblyopic eyes was found when compared with normal eyes, whereas no significant relationship between OCT and PERG parameters was discovered. The function of IPL could be accessed by the ERG [14] (oscillatory potential overlaps in B waves, reflecting IPL synaptic activation). The current study proves the structural change of IPL that could be helpful to find the relationship of retinal function and morphology changes in amblyopic eyes.

Rotruck et al. [15] did a study of normative macular data in children ages 0-5 years. In patients < 18 months old, an inverse relationship was found between age and mean thickness of the perifovea for the GCL, while in patients ≥ 18 months, no relationship was found between them. Huynh et al. [5] have proposed that arrest of normal postnatal changes in amblyopic eyes could affect the normal maturation of the macular. Our finding of the decreased GCL or IPL thickness in inner subfields could be helpful for understanding the pathological changes in the amblyopic eyes. However, as a cross-section study, it is impossible to determine the causal relationship; further study with a larger sample size and a longer observation period is needed.

In the current study, two of the four quadrants of the GCL thickness and three of the four quadrants of the IPL thickness in the peripheral macular area were significantly decreased in fellow eyes as compared to control eyes. This is consistent with some studies [16, 17] that reported both eyes in children with unilateral amblyopia had deviations when compared with normal children. Chang-Bing et al. [18] reported that anisometropic amblyopia resulted in both monocular and interocular dysfunctions. This functional imbalance between amblyopic eye and fellow eye may cause irreversible changes which affect the visual pathway of both eyes. This is also the reason why we compared amblyopic eyes with normal eyes instead of fellow eyes.

Several studies have examined macular thickness with OCT in amblyopia in the past decade. In this study, there was no significant difference noted in TMT between amblyopic and control eyes. Similar findings were reported in some studies [5, 19–21], whereas Yi et al. [4] reported that the fovea of amblyopic eye tends to be thicker than that of the normal fellow eye, while the inner and outer macula tend to be thinner. The difference in the results of these studies may be due to the experimental design, ethnicity, or refractive error. Furthermore, some of these studies were performed with time domain OCT (TD-OCT), which has a resolution of 10 *μ*m axially. In addition, most of the differences in thicknesses reported were even less than 10 *μ*m, which made the outcomes of these studies unreliable. Besides, because of the low resolution of TD-OCT, these studies were limited to TMT.

Unlike some previous studies, we adopted the built-in automatic layer segmentation software of the Spectralis OCT, which made it possible to compare the results of different laboratories with the same platform. Besides, this study analyzed the average thickness data of each area of the macula to avoid the possible deviation caused by measuring only one point in each direction in some studies [10, 11].

Limitations of our study should be discussed: The sample size of anisometropic amblyopic eyes was relatively small, though it was bigger than that in previous studies. Besides, for the reason of children's cooperation, the thickness of each layer in the outer ring was not analyzed in order to ensure the reliability of the data.

## 5. Conclusions

The SD-OCT data revealed differences in the thicknesses of some macular inner retinal layers in both eyes of children with anisometropic amblyopia compared with those with emmetropia, indicating that structural changes might exist in the retina of children with amblyopia. Further studies, including a larger area in the macula, with more numbers of patients and correlation with retinal function are required to confirm these findings.

## Figures and Tables

**Table 1:** Demographic and clinical description.

	Amblyopic eyes	Fellow eyes	Control eyes	P1	P2
Number	37	37	57		
Male/female	16/21	16/21	25/32		
Age	7.43 ± 2.62	7.43 ± 2.62	8.05 ± 1.33	0.19	0.19
Axial length (mm)	22.41 ± 1.76	22.57 ± 1.39	22.96 ± 0.67	0.08	0.12
LogMAR	0.40 ± 0.22	0.06 ± 0.08	−0.02 ± 0.03	<0.01^a^	<0.01^a^
Refractive error (*D*)	+1.52 ± 3.42	+0.21 ± 1.50	−0.02 ± 0.16	<0.01^a^	<0.01^a^

LogMAR: logarithm of the minimum angle of resolution; P1: amblyopic eyes vs. control eyes; P2: fellow eyes vs. control eyes. ^a^A statistically significant difference between amblyopic, fellow, and control eyes (*p* < 0.05).

**Table 2:** The mean values of the total macular thickness (in *μ*m).

	Amblyopic eyes	Fellow eyes	Control eyes	P1	P2
	(mean ± SD)	(mean ± SD)	(mean ± SD)		
C1	254.83 ± 23.73	253.48 ± 21.51	255.91 ± 18.87	0.81	0.56
S3	339.64 ± 16.70	344.86 ± 28.21	342.84 ± 12.73	0.3	0.63
I3	330.81 ± 19.66	335.78 ± 20.98	335.71 ± 13.01	0.15	0.98
T3	322.72 ± 18.59	321.54 ± 15.89	327.52 ± 12.35	0.14	0.04^a^
N3	333.11 ± 24.19	336.27 ± 16.48	340.70 ± 14.13	0.06	0.17

P1: amblyopic eyes vs. control eyes; P2: fellow eyes vs. control eyes; SD: standard deviation. ^a^A statistically significant difference between amblyopic, fellow, and control eyes (*p* < 0.05).

**Table 3:** The mean values of individual inner retinal layer thickness (in *μ*m).

	Amblyopic eyes	Fellow eyes	Control eyes	P1	P2
	(mean ± SD)	(mean ± SD)	(mean ± SD)		
C1 RNFL	11.54 ± 3.00	11.00 ± 2.52	11.11 ± 2.33	0.46	0.84
S3 RNFL	24.05 ± 3.37	25.84 ± 10.38	23.02 ± 2.86	0.11	0.12
I3 RNFL	23.35 ± 3.42	26.38 ± 23.65	23.44 ± 2.78	0.89	0.46
T3 RNFL	17.30 ± 2.47	16.65 ± 1.25	16.60 ± 1.29	0.08	0.85
N3 RNFL	20.11 ± 2.87	20.03 ± 3.35	19.88 ± 2.32	0.67	0.80
C1 GCL	15.24 ± 6.26	14.27 ± 7.01	14.05 ± 3.91	0.26	0.85
S3 GCL	51.43 ± 4.11	51.46 ± 4.60	54.53 ± 4.14	<0.01^a^	<0.01^a^
I3 GCL	50.30 ± 5.58	51.16 ± 4.66	51.74 ± 7.50	0.32	0.65
T3 GCL	46.16 ± 4.43	45.24 ± 5.55	49.18 ± 3.55	<0.01^a^	<0.01^a^
N3 GCL	48.19 ± 6.57	49.73 ± 5.36	51.56 ± 4.00	0.01^a^	0.06
C1 IPL	19.27 ± 4.95	19.46 ± 5.02	19.02 ± 3.19	0.76	0.60
S3 IPL	40.11 ± 2.32	40.30 ± 3.10	41.54 ± 2.44	0.01^a^	0.03^a^
I3 IPL	39.38 ± 3.62	40.19 ± 2.42	40.88 ± 2.47	0.02^a^	0.19
T3 IPL	39.73 ± 3.54	39.22 ± 3.89	40.72 ± 2.30	0.10	0.04^a^
N3 IPL	39.62 ± 4.01	40.70 ± 3.16	42.16 ± 2.94	<0.01^a^	0.03^a^
C1 INL	17.78 ± 5.50	16.65 ± 5.43	15.63 ± 4.45	0.04^a^	0.32
S3 INL	41.41 ± 4.16	41.43 ± 6.47	41.42 ± 2.97	0.98	0.99
I3 INL	41.57 ± 5.17	41.43 ± 4.61	40.88 ± 3.60	0.48	0.52
T3 INL	38.81 ± 4.88	37.76 ± 4.63	38.96 ± 3.15	0.87	0.17
N3 INL	40.54 ± 5.13	41.54 ± 5.54	39.98 ± 3.70	0.54	0.11

P1: amblyopic eyes vs. control eyes; P2: fellow eyes vs. control eyes; SD: standard deviation. ^a^A statistically significant difference between amblyopic, fellow, and control eyes (*p* < 0.05).

## Data Availability

The data used to support the findings of this study are available from the corresponding author upon request.
